# Integrative Genomics Sheds Light on Evolutionary Forces Shaping the Acidithiobacillia Class Acidophilic Lifestyle

**DOI:** 10.3389/fmicb.2021.822229

**Published:** 2022-02-15

**Authors:** Carolina González-Rosales, Eva Vergara, Mark Dopson, Jorge H. Valdés, David S. Holmes

**Affiliations:** ^1^Center for Bioinformatics and Genome Biology, Centro Ciencia & Vida, Fundación Ciencia & Vida, Santiago, Chile; ^2^Center for Genomics and Bioinformatics, Faculty of Sciences, Universidad Mayor, Santiago, Chile; ^3^Centre for Ecology and Evolution in Microbial Model Systems, Linnaeus University, Kalmar, Sweden; ^4^Center for Bioinformatics and Integrative Biology, Facultad de Ciencias de la Vida, Universidad Andrés Bello, Santiago, Chile; ^5^Facultad de Medicina y Ciencia, Universidad San Sebastián, Santiago, Chile

**Keywords:** acidophiles, pH homeostasis, extremophiles, acid mine drainage (AMD), evolution, comparative genomics

## Abstract

Extreme acidophiles thrive in environments rich in protons (pH values <3) and often high levels of dissolved heavy metals. They are distributed across the three domains of the Tree of Life including members of the Proteobacteria. The Acidithiobacillia class is formed by the neutrophilic genus *Thermithiobacillus* along with the extremely acidophilic genera *Fervidacidithiobacillus*, *Igneacidithiobacillus*, *Ambacidithiobacillus*, and *Acidithiobacillus*. Phylogenomic reconstruction revealed a division in the Acidithiobacillia class correlating with the different pH optima that suggested that the acidophilic genera evolved from an ancestral neutrophile within the Acidithiobacillia. Genes and mechanisms denominated as “first line of defense” were key to explaining the Acidithiobacillia acidophilic lifestyle including preventing proton influx that allows the cell to maintain a near-neutral cytoplasmic pH and differ from the neutrophilic Acidithiobacillia ancestors that lacked these systems. Additional differences between the neutrophilic and acidophilic Acidithiobacillia included the higher number of gene copies in the acidophilic genera coding for “second line of defense” systems that neutralize and/or expel protons from cell. Gain of genes such as hopanoid biosynthesis involved in membrane stabilization at low pH and the functional redundancy for generating an internal positive membrane potential revealed the transition from neutrophilic properties to a new acidophilic lifestyle by shaping the *Acidithiobacillaceae* genomic structure. The presence of a pool of accessory genes with functional redundancy provides the opportunity to “hedge bet” in rapidly changing acidic environments. Although a core of mechanisms for acid resistance was inherited vertically from an inferred neutrophilic ancestor, the majority of mechanisms, especially those potentially involved in resistance to extremely low pH, were obtained from other extreme acidophiles by horizontal gene transfer (HGT) events.

## Introduction

Microbial cells present a variety of genetic mechanisms allowing them to manage stressful situations such as changes in temperature, pH values, and oxidative stress (Li et al., 2011). Organisms with an acidic optimal growth pH are termed “acidophiles,” and this classification has been further divided into moderate acidophiles with a pH optimum ≤5 and extreme acidophiles that grow optimally at pH ≤3. To survive and grow in low pH environments, acidophiles must maintain a near-neutral cytoplasm when faced with an external to internal proton gradient across the cytoplasmic membrane up to 10^5^-fold (Slonczewski et al., 2009; Zammit and Watkin, 2016; Quatrini and Johnson, 2018). Different pH homeostasis mechanisms have been proposed to explain the acidophilic resistance including some that are shared with neutrophiles. These are (i) proton export pumps and antiporters (Michels and Bakker, 1985); (ii) cytoplasmic buffering *via* an overproduction of alkaline amino acids (Parro et al., 2007); (iii) proton consuming reactions such as glutamate decarboxylase (Mangold et al., 2013); (iv) alterations in the membrane structure to reduce fluidity by the inclusion of hopanoids (Mykytczuk et al., 2010); (v) reduction in the outer membrane permeability *via* porins using polyamines such as spermidine (Samartzidou et al., 2003); and (vi) pH homeostatic systems such as an internal positive membrane potential thought to be generated by potassium ions (Buetti-Dinh et al., 2016). However, as pH homeostatic mechanisms are often shared between neutrophiles and acidophiles, those most important to mediating homeostasis in highly acidic conditions are not always apparent.

Low pH mitigation mechanisms have been investigated in acidophiles (Baker-Austin and Dopson, 2007; Li et al., 2011; Ullrich et al., 2016a; Colman et al., 2018; Vergara et al., 2020; Chen et al., 2021; Mayer et al., 2021; Sriaporn et al., 2021). These studies show that horizontal gene transfer (HGT), gene mutation, and gene loss trajectories in evolution allow adaptation and survival of prokaryotes in extreme conditions (Foster, 2004; Ullrich et al., 2016a; Zhang et al., 2017; Vergara et al., 2020). Extensive HGT events are also documented in acidophiles such as *Sulfolobus islandicus* (Whitaker et al., 2005), *Leptospirillum* (Simmons et al., 2008; Vergara et al., 2020), and the extremophilic red alga *Galdieria sulphuraria* (Schonknecht et al., 2013; Colman et al., 2018) that acquired genes by HGT from bacteria and archaea to be able to thrive in thermophilic, acidic, and metal-rich environments. To cope with the high acidity, *G. sulphuraria* lowers the proton permeability of its plasma membrane by having a single copy of a voltage-gated ion channel gene compared to three copies in the neutrophilic *Cyanidioschyzon merolae* from which it diverged one billion years ago (Schonknecht et al., 2013). However, the events that led to the evolution of acidophiles and their role in the generation of acidic habitats are underexplored.

The Acidithiobacillia is a newly recognized class of Proteobacteria at the root of the Betaproteobacteria/Gammaproteobacteria division (Williams and Kelly, 2013). Two families make up this class, family I comprising the acidophilic *Acidithiobacillaceae* and the neutrophilic family II *Thermithiobacillaceae*. Due to their acidophilic nature, the *Acidithiobacillaceae* are largely recalcitrant to standard genetic manipulation (Inaba et al., 2018; Jung et al., 2021), and consequently, the use of bioinformatics approaches advances our understanding of the biology of these extremophiles. The research of Moya-Beltrán et al. (2021) describes four *Acidithiobacillaceae* genera (Nuñez et al., 2016), namely, (i) sulfur oxidizing *Ambacidithiobacillus* that includes the recently described species *Am. sulfuriphilus* (ex *Acidithiobacillus sulfuriphilus*) (Falagán et al., 2019); (ii) *Igneacidithiobacillus* including species *I. copahuensis* (Moya-Beltrán et al., 2021), *Candidatus I. taiwanensis* (Moya-Beltrán et al., 2021), and *Candidatus I. yellowstonensis* (Moya-Beltrán et al., 2021); (iii) *Fervidacidithiobacillus* with *F. caldus* (ex-*Acidithiobacillus caldus*) (Hallberg and Lindström, 1994; Valdes et al., 2009; You et al., 2011; Zhang et al., 2016b); and (iv) the *Acidithiobacillus* genus formed by *A. thiooxidans* (Waksman and Joffe, 1922; Valdes et al., 2011; Travisany et al., 2014; Yin et al., 2014; Zhang et al., 2016a,^2018^; Quatrini et al., 2017; Camacho et al., 2020), *A. albertensis* (Bryant et al., 1983; Castro et al., 2017), *Acidithiobacillus* sp. SH (Kamimura et al., 2018) plus the iron/sulfur oxidizers *A. ferrooxidans* (Temple and Colmer, 1951; Valdés et al., 2008; Chen P. et al., 2015; Yan et al., 2015; Kucera et al., 2016; Ulloa et al., 2019), *A. ferridurans* (Hedrich and Johnson, 2013; Miyauchi et al., 2018), *A. ferrivorans* (Hallberg et al., 2010; Liljeqvist et al., 2011; Talla et al., 2014; Tran et al., 2017), and *A. ferriphilus* (Falagán and Johnson, 2016) represented by *A. ferrooxidans* BY0502 for which phylogenetic and nucleotide identity analysis is suggested to be reclassified as an *A. ferriphilus*-like species (González et al., 2016; Fariq et al., 2019).

Genome sequences are available for many of the acidophilic species (Neira et al., 2020) including *A. ferrooxidans* (Valdés et al., 2008; Chen P. et al., 2015; Yan et al., 2015; Kucera et al., 2016; Ulloa et al., 2019), *A. ferrivorans* (Liljeqvist et al., 2011; Talla et al., 2014; Tran et al., 2017), *A. ferridurans* (Hedrich and Johnson, 2013; Miyauchi et al., 2018), *A. ferrianus* (Norris et al., 2020), *A. thiooxidans* (Valdes et al., 2011; Travisany et al., 2014; Yin et al., 2014; Zhang et al., 2016a,^2018^; Quatrini et al., 2017; Camacho et al., 2020), *F. caldus* (Valdes et al., 2009; You et al., 2011; Zhang et al., 2016b), and *Am. sulfuriphilus* (Falagán et al., 2019) along with the neutrophile *Thermithiobacillus tepidarius* (Boden et al., 2016).

Three populations of uncultivated hot spring *Acidithiobacillus* strains and ten publicly available genomes were analyzed for the presence or absence of predicted genes for acid resistance, identifying a similar number of gene copies encoding for K^+^ transporters, deiminases/deaminase group and adenosine deaminase shared between genomes, and a difference in the number of amino acid decarboxylases, Na^+^/H^+^ antiporters, and plasma membrane proton-efflux P-type ATPases (Sriaporn et al., 2021). In addition, the mechanisms of resistance to low temperature and pH from an extreme microbial community where *A. ferrivorans* was the dominant member revealed an adaptation to low temperature by the presence trehalose synthase pathways, oxidative stress pathways, cold shock proteins, and genes encoding for biofilm formation (González et al., 2014; Liljeqvist et al., 2015). This study investigates the genes present in the Acidithiobacillia class by phylogenomic and comparative genomic analyses deepening our understanding of how the acidophilic genera *Fervidacidithiobacillus*, *Igneacidithiobacillus*, *Ambacidithiobacillus*, and *Acidithiobacillus* obtained genes coding for pH homeostasis that allows them to survive at extremely low pH values of ≤3.

## Materials and Methods

### Genome Selection and Representative Selection

Thirty-seven permanent draft or complete genomes from the Acidithiobacillia class were downloaded from NCBI with those genomes passing CheckM (Parks et al., 2015) quality control for completeness (>90%) and lack of contamination (<10%) retained. The Acidithiobacillia class conserved core genome was identified using the GET_Homologues (Contreras-Moreira and Vinuesa, 2013) software suite, selecting conserved proteins with at least 50% identity and alignment coverage. Core protein families were aligned using MAFFT (Katoh, 2002; Katoh and Standley, 2013; Katoh et al., 2019) with L-INS-i iterative refinement. Alignments were masked to remove unreliable aligned regions with GBLOCK (Castresana, 2000) followed by concatenation of families. A maximum likelihood tree was constructed based on concatenated alignment of core genes using IQ-TREE (Nguyen et al., 2015; Hoang et al., 2018) with 1,000 replicates and the best-fit model predicted by IQ-TREE (Nguyen et al., 2015; Kalyaanamoorthy et al., 2017) according to Bayesian information criterion and the *T. tepidarius* DSM 3134 genome as an outgroup for the *Acidithiobacillaceae* (Boden et al., 2016). The phylogenomic tree was visualized with iTOL6 (Letunic and Bork, 2021).

A representative genome was selected from each clade of core genomes according to the following criteria (Supplementary Figure 1): (i) using the type strain genome if available and complete; (ii) if this was unavailable or a draft, a strain with a complete genome was selected; and (iii) a strain genome sequence was selected according to the quality as calculated by CheckM.

### Protein Family Prediction

Conserved orthologous proteins were selected based on the classification of all protein-coding genes in protein families. The selected proteins were sorted using the GET_Homologues (Contreras-Moreira and Vinuesa, 2013) software suite, with BLAST (Altschul, 1997) and orthoMCL (Fischer et al., 2011) programs. Protein families were constructed using a 50/50 rule of 50% of identity and 50% of alignment coverage (Altschul et al., 1990; Altschul, 1997; Snipen and Ussery, 2010) and each protein was then assigned to one protein family. The protein families were classified in core (proteins shared by all strains), dispensable (proteins assigned to some strains), and unique (proteins assigned to only a single strain) genomes according to their distribution (Supplementary Data Sheet 1). The sum of all three groups form the pangenome, i.e., the union of the genomes under consideration (Tettelin et al., 2005; Snipen and Ussery, 2010).

### Selection of Acid Resistance Genes

Genes and protein sequences that were either predicted or experimentally validated to be involved in low pH responses in extreme acidophiles were extracted from the literature (Gassel et al., 1998; Foster, 2004; Zhou et al., 2010; Krulwich et al., 2011; Mangold et al., 2013; De Biase and Lund, 2015; Mühling et al., 2016; Vergara et al., 2020). The list of potential genes was extended by keyword literature searches in Google Scholar and species description journals (e.g., International Journal of Systematic and Evolutionary Microbiology). Additionally, the JGI pH metadata and NCBI’s BIOSAMPLE database were used to collect gene and protein information linked to low pH environments. The collected assemblage of potential acid resistance gene/protein sequences was used to formulate Blast searches against Acidithiobacillia genomes using a minimal *E*-value cutoff of 1e^–5^ (Supplementary Figure 1).

### Protein Properties

Prediction of transmembrane regions in protein sequences was carried out using TMHMM (Sonnhammer et al., 1998; Krogh et al., 2001) and TMPRED (Chen et al., 2002). Signal peptide and subcellular localization predictions were made with SignalP 5.0 (Almagro Armenteros et al., 2019), PSORTb (Yu et al., 2010), and CELLO (Yu et al., 2006). Prediction of lipoprotein signals, search of lipobox, plus identification of conserved sequences, motifs, and domains were made with LipoP Server (Juncker et al., 2003) and WebLogo (Schneider and Stephens, 1990; Crooks et al., 2004) using AliView (Larsson, 2014) and MAFFT (Katoh, 2002; Katoh and Standley, 2013; Katoh et al., 2019) as alignment tools.

### Mobile Element Analysis and Horizontal Gene Transfer Prediction

Mobile genetic elements as insertion sequences in Acidithiobacillia genomes were predicted with ISsaga (Varani et al., 2011) of ISFINDER (Kichenaradja et al., 2010; Varani et al., 2011) and TnpPred (Riadi et al., 2012). Potential HGT events were predicted by HGTector (Zhu et al., 2014). Genes and pathways related to acid resistance were analyzed to identify mobile genetic elements as transposases, integrases, or phage elements in close genomic context using IslandViewer (Bertelli et al., 2017), Artemis (Carver et al., 2012), MAUVE (Darling et al., 2004, 2010), and STRING (von Mering et al., 2005; Szklarczyk et al., 2019).

### Phylogenetic Tree Construction of Acid Resistance Genes

Trees of acid resistance proteins were constructed for proteins identified in the Acidithiobacillia class and orthologs from other microorganisms to propose a common or different evolutionary origin. Orthologous proteins from acid resistance genes were obtained by selection of best hits from BlastP comparison of Acidithiobacillia acid resistance genes versus the NCBI database. Phylogenies were constructed using acid resistance genes from Acidithiobacillia plus orthologous proteins from NCBI, which were aligned with MAFFT using L-INS-i iterative refinement (Katoh, 2002; Katoh and Standley, 2013; Katoh et al., 2019) and IQ-TREE (Nguyen et al., 2015; Kalyaanamoorthy et al., 2017; Hoang et al., 2018) for prediction of best suited model and phylogenetic construction. Visualization of the trees was with Figtree^1^ and iTOL6 (Letunic and Bork, 2021).

## Results and Discussion

### General Features of Acidithiobacillia Pangenome

**FIGURE 1:**
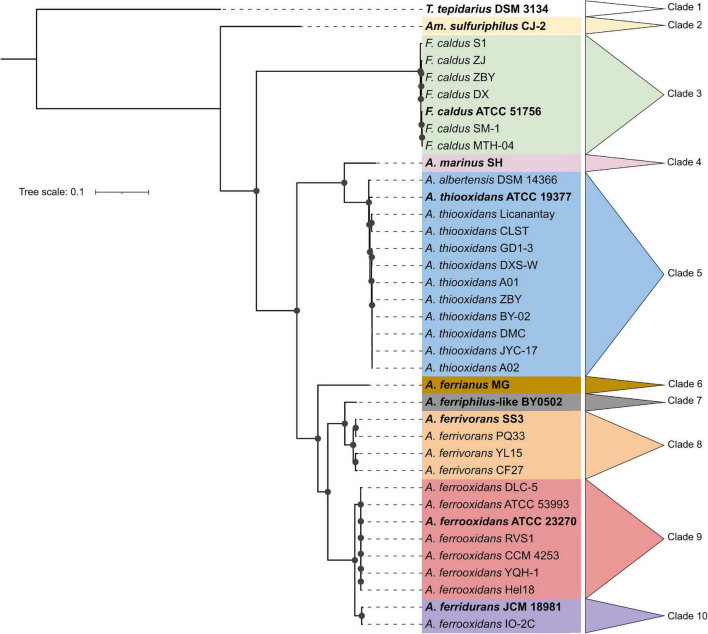
Maximum likelihood phylogenetic tree showing the neutrophilic ancestral origins of the Acidithiobacillia class. The bold genomes were selected to represent the ten clades from Acidithiobacillia class core tree, including *T. tepidarius* DSM 3134 for the neutrophilic *Thermithiobacillus* genus and nine genomes for the acidophilic genera *Ambacidithiobacillus* (*Am*.), *Fervidacidithiobacillus* (*F*.), and *Acidithiobacillus* (*A*.). The tree was constructed using 440 conserved proteins using 1,000 replicates. Bootstraps values ≥60% are represented by black dots on the nodes. The scale bar represents 0.1 amino acid substitution per position.

Thirty-seven Acidithiobacillia class genomes met the CheckM quality criteria while the pangenome analysis showed a core genome of 440 protein families (Supplementary Data Sheet 1). A maximum likelihood phylogenetic tree of Acidithiobacillia core concatenated proteins showed ten clades, separating the neutrophilic *T. tepidarius* from the acidophilic genera *Ambacidithiobacillus*, *Fervidacidithiobacillus*, and *Acidithiobacillus* (Figure 1). The acidophiles were further grouped into nine clades, of which two represented different genera (clades 2 and 3); meanwhile, clades 4–10 grouped species from the *Acidithiobacillus* genus, which is divided into clades according to their capacity to obtain energy such as sulfur oxidizers (clades 4 and 5) and iron/sulfur oxidizers (clades 6–10). While the association between the *Acidithiobacillaceae* and *Thermithiobacillaceae* families was consistent with previous analyses (Williams et al., 2010; Williams and Kelly, 2013; Boden et al., 2016; Moya-Beltrán et al., 2021), the tree also suggested a need for deeper phylogenomic analysis such as for the *Acidithiobacillus* clade, questioning if this clade includes more than a single genus according to their energy properties. Representative genomes from the ten clades of the core tree were selected for this study with their accession numbers and isolation data summarized in Table 1.

**TABLE 1:** Strains, genome accession, and features of the selected representatives of the Acidithiobacillia class.

	Genome state and accession number	Source	Size (Mbp)	G+C (%)	#CDS	# contigs/# scaffolds	References
*T. tepidarius* 3134^T^	Draft AUIS00000000	United Kingdom	2.96	66.8	2903	0/43	Boden et al., 2016
*Am. sulfuriphilus* CJ-2^T^	Draft RIZI01000000	United Kingdom	2.82	61.5	2738	0/195	Falagán et al., 2019
*F. caldus* ATCC 51756^T^	Complete CP005986	United Kingdom	2.78	61.7	2681	1/0	Valdes et al., 2009
Megaplasmid mpAca1.1	CP005987	United Kingdom	0.17	57.4	208	1/0	
Plasmid pACA1.1	CP005988	United Kingdom	0.03	59.4	33	1/0	
Plasmid pACA1.2	CP005989	United Kingdom	0.01	50.3	11	1/0	
*A.* marinus SH^T^	Draft MXAV01000000	Japan	2.9	54.3	2828	0/65	Kamimura et al., 2018
*A. thiooxidans* ATCC 19377^T^	Complete CP045571.1	United Kingdom	3.42	53	3388	1/0	^a^, Valdes et al., 2011; Camacho et al., 2020
*A. ferrianus* MG^T^	Draft WNJL01000000	Greece	3.17	58.2	3020	0/90	Norris et al., 2020
“*A. ferriphilus*-like BY0502” (formerly *A. ferrooxidans* BY0502)	Draft LVXZ01000000	China	2.98	56.8	2816	0/295	^b^
*A. ferrivorans* SS3	Complete CP002985	Russia	3.21	56.6	3093	1/0	Liljeqvist et al., 2011
*A. ferridurans* JCM 18981	Complete AP018795	Japan	2.92	58.4	3026	1/0	Miyauchi et al., 2018
*A. ferrooxidans* ATCC 23270^T^	Complete CP001219	United States	2.98	58.8	3147	1/0	Valdés et al., 2008

*^a^Wang et al. unpublished. Acidithiobacillus thiooxidans genome sequencing and assembly. Submitted (OCT-2019) to EMBL/GenBank/DDBJ databases.*

*^b^Zhou et al. unpublished. Acidithiobacillus ferrooxidans genome sequencing and assembly. Submitted (APR-2016) to the EMBL/GenBank/DDBJ databases.*

### Acidithiobacillia Acid Resistance Mechanisms

Genes with predicted or experimental evidence for acid resistance mechanisms were searched in the Acidithiobacillia class using a bioinformatic pipeline (Supplementary Figure 1) that generated the list in Supplementary Data Sheet 2. The acid resistance genes were further labeled into first and second line of defense, according to the classification system proposed by the research of Vergara et al. (2020), which proposes that genes involved in the prevention of the entry of proton into the cell correspond to the “first line of defense,” and those related to neutralization or expulsion of protons inside the cell belong to the “second line of defense.”

### First Line of Defense of Acidophilic Lifestyle in the Acidithiobacillia

**FIGURE 2:**
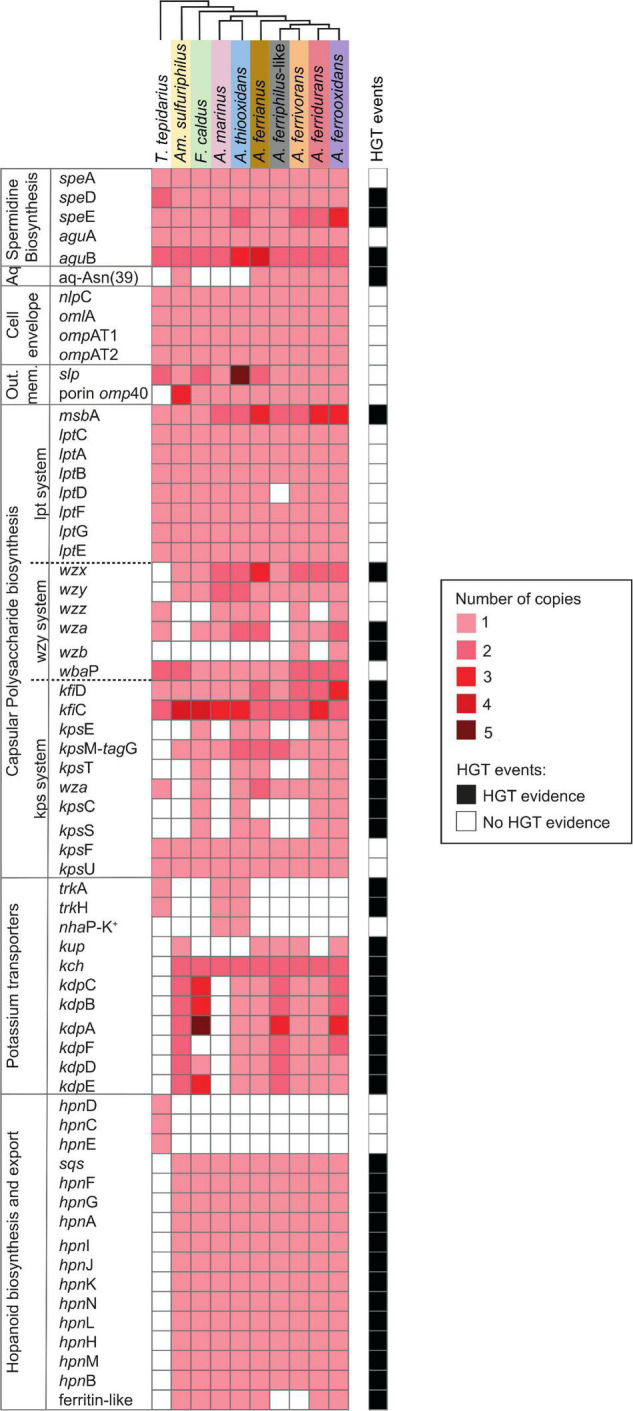
Heatmap of presence (red) and absence (white) of genes involved in the first line of Acidithiobacillia class acid resistance including spermidine biosynthesis; aquaporine (Aq) with an asparagine residue (Asn39); cell envelope; outer membrane proteins (Out. mem.); capsular polysaccharide biosynthesis with pathways Lpt-, Wzy-, and Kps-systems; potassium transporters; and hopanoid biosynthesis plus export. The color intensity represents higher gene copy number per genome. The presence of genes (black; right-hand side) denotes HGT events by genome context with mobile genetic elements, phylogenetic trees, or if it was signaled as part of an HGT event by HGTector (Zhu et al., 2014). *T. tepidarius*, *Thermithiobacillus tepidarius* DSM 3134; *Am. Sulfuriphilus*, *Ambacidithiobacillus sulfuriphilus* CJ-2; *F. caldus*, *Fervidacidithiobacillus caldus* ATCC 51756; *A. marinus*, *Acidithiobacillus marinus* SH; *A. thiooxidans*, *A. thiooxidans* ATCC 19377; *A. ferrianus*, *A. ferrianus* MG; *A. ferriphilus*-like, *A. ferriphilus*-like BY0502; *A. ferrivorans*, *A. ferrivorans* SS3; *A. ferridurans*, *A. ferridurans* JCM 18981; and *A. ferrooxidans*, *A. ferrooxidans* ATCC 23270 (shaded colors correspond to those used in Figure 1).

Genes related to the first line of defense involved in the prevention of entry of protons into the cell, membrane rigidity, reduced membrane permeability to protons, and maintenance of cellular integrity are summarized in Figure 2.

**FIGURE 3:**
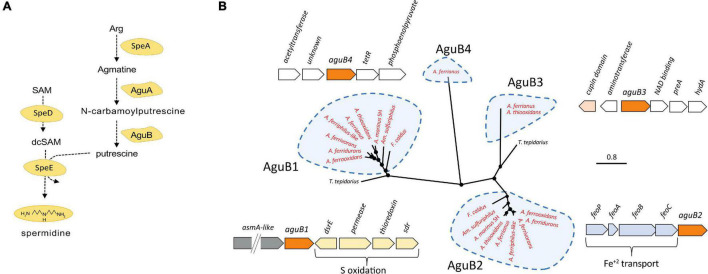
**(A)** Putrescine and spermidine biosynthesis pathway. **(B)** Unrooted phylogenetic tree of Acidithiobacillia class *agu*B amino acid sequences and their best hits in the NCBI databases plus their genomic context in the *Acidithiobacillaceae* (*agu*B in orange color). Sequences of *agu*B group in four clades denominated *agu*B1–*agu*B4. Acidophilic sequences are highlighted in red. Filled circles at the nodes indicate bootstrap support ≥60%. The scale bar represents 0.8 amino acid substitution per site.

The putrescine biosynthesis pathway was identified in the Acidithiobacillia class including genes *spe*A, *agu*A, and one or more copies of *agu*B (Figure 3A). Acidophilic genomes from the *Acidithiobacillaceae* family present several copies of *agu*B clustered in four clades *agu*B1–*agu*B4 (Figure 3B). The *agu*B1 and *agu*B3 clades associated *agu*B from acidophiles with the neutrophile *T. tepidarius* although the *agu*B3 genome context in the acidophiles displayed a cupin domain gene with high similarity to copies in the acidophiles *Acidocella* and *Acidiphilium*. In addition, *agu*B3 genes were predicted to result from HGT in the *Acidithiobacillus* genus. The clade of acidophile *agu*B2 genes were downstream from an *feo*ABC operon related to iron management (Osorio et al., 2008).

**FIGURE 4:**
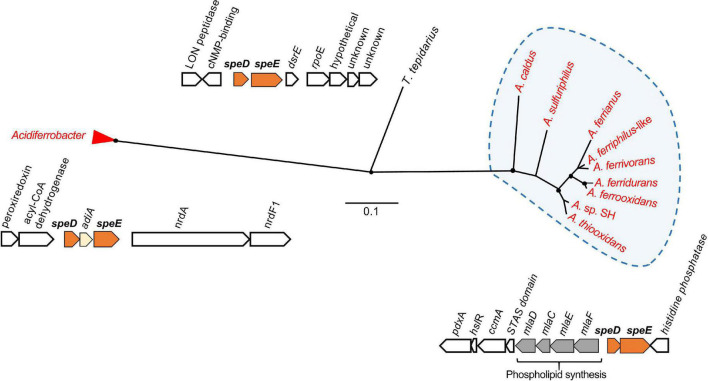
Unrooted phylogenetic tree of concatenated amino acid sequences of *spe*D and *spe*E in the Acidithiobacillia and their best hits in the NCBI databases. *Acidithiobacillaceae* sequences group in a clade from neutrophilic *T. tepidarius*, and had highest similarity to homologs in extreme acidophiles of the *Acidiferrobacter* group. Acidophilic sequences are highlighted in red. Filled circles at the nodes indicate bootstrap support ≥60%. The scale bar represents 0.1 amino acid substitution per site.

Putrescine is required for spermidine synthesis (Figure 3A), which protects the cell against acid stress by cytoplasmic buffering (Rhee et al., 2007; Kanjee and Houry, 2013; Vergara et al., 2020) such as in *F. caldus* (Mangold et al., 2013). S-adenosylmethionine decarboxylase (*spe*D) and spermidine synthase (*spe*E) are essential for the biosynthesis of spermidine (Xie et al., 1989), which are significantly upregulated in acidic environments (Li et al., 2012). This gene cluster was conserved in the Acidithiobacillia, separating into two clades between the neutrophile *T. tepidarius* and acidophiles of *Acidithiobacillaceae* with different genome contexts. The acidophile spermidine synthesis genes were downstream of *mla*DCEF (Figure 4); the latter acts as an ABC transporter driving translocation of phospholipids between the inner and outer membrane of Gram-negative bacteria (Hughes et al., 2019; Chi et al., 2020). The *mla*D outer membrane lipid and *mla*C phospholipid ABC transporter maintain outer membrane integrity in osmotic stress conditions in the halotolerant acidophile *Acidihalobacter prosperus* (Malinverni and Silhavy, 2009; Dopson et al., 2017; Khaleque et al., 2019). Phylogenetic analysis of concatenated *spe*D-*spe*E genes showed similarity to the extreme acidophile *Acidiferrobacter* (Figure 4). The close genomic context between *spe*DE and the *mla*DCEF gene complex observed in acidophiles highlights the relevance of these genes for membrane maintenance as a stress response.

An aquaporin AqpF was conserved in the *Ambacidithiobacillus* and *Acidithiobacillus* genera (Supplementary Figure 2), including three copies in *A. ferrivorans*. The aquaporin AqpF with an asparagine residue (Asn39) is proposed to be involved in enhancing the capability for proton-blocking in extreme acidophiles (Duarte et al., 2009). This difference was identified in the Acidithiobacillia where the AqpF in the neutrophiles *T. tepidarius* lacked this residue in contrast to AqpF in acidophiles *Am. sulfuriphilus* and *Acidithiobacillus* iron oxidizers. Aquaporin genes identified in *A. ferrianus* and *A. ferriphilus*-like presented signals of HGT events with the extreme acidophile *Acidihalobacter*-like as the potential donor.

Cell envelope *nlp*C, *oml*A, *omp*AT1, and *omp*AT2 genes were conserved in the Acidithiobacillia class with a single copy per genome that is upregulated in response to low pH conditions in *A. ferrooxidans* (Chao et al., 2008). The gene *omp*40 was exclusively identified in acidophilic members of the Acidithiobacillia including three copies in *Am. sulfuriphilus* CJ-2. The Omp40 is a major outer membrane ion channel protein that increases in expression in response to low pH and phosphate starvation in *F. caldus* and *A. ferrooxidans* (Amaro et al., 1991; Mangold et al., 2013; Hu et al., 2020), suggesting that it controls proton influx across the outer membrane (Guiliani and Jerez, 2000; Baker-Austin and Dopson, 2007). A further cell envelope gene *slp* prevents the flux of organic acids across the outer membrane counteracting their toxic effect in acidophiles (Alexander et al., 1987; Mates et al., 2007; Vergara et al., 2020) and ameliorating low pH stress conditions in *Escherichia coli* (Mates et al., 2007). The *slp* genes contain a characteristic lipobox motif with an Asn amino acid residue in the *+*2 position, indicative that the protein is located at the inner membrane, and a different residue if Slp protein is exported (Zückert, 2014; Vergara et al., 2020). The *slp* gene was present in all Acidithiobacillia genomes (including several copies in some species; Supplementary Figure 3A) and was predicted to be exported. Phylogenetic analysis grouped *slp* into three clades (Supplementary Figure 3B) with the neutrophilic *slp* genes separated from those in the acidophile genomes.

Capsule polysaccharides constitute a mechanical defense layer (Feng et al., 2015) that acts as a protective barrier around the cell (Li et al., 2011), preventing influx of protons (Baker-Austin and Dopson, 2007; Mykytczuk et al., 2010) and other harmful external factors (Plante, 2000; Feng et al., 2015; Hu et al., 2020). The *A. thiooxidans* capsule became significantly thicker at the sub-optimal acid pH 0.8, and at super acid pH 0.4 began to fade, owing to that extreme acid stress became an unbearable threat to the cell survival (Feng et al., 2015, 2019). Three Acidithiobacillia capsule polysaccharide pathways were identified. These were the Lpt (Ma et al., 2008; Renzi et al., 2016), Wzy-dependent (Yuan et al., 2013; Barahona et al., 2014; Wen and Zhang, 2015; Renzi et al., 2016; Oetiker et al., 2018), and Kps (Barton, 2005; Vera et al., 2013; Nzakizwanayo et al., 2015) systems (Figure 2). The Lpt system was conserved in the Acidithiobacillia, with additional copies of the ABC transporter *msb*A in the *Acidithiobacillaceae* and absence of *lpt*D in the *A. ferriphilus*-like genome (probably due to its draft state). The *Acidithiobacillaceae* family encode genes for the Wzy system such as *wzx* for flippases that transport capsule polysaccharides across the cytoplasmic membrane along with the accessory genes *wzz*, *wza*, *wzb*, and *wba*P. The accessory genes *wzx, wzz*, and *wza* were predicted to result from HGT events from potential Proteobacteria, *Acidihalobacter*-like, and Bacteroidetes donors. The Kps system includes KfiC and KfiD required for polysaccharide biosynthesis and the export pathway consisting of the Wza integral outer membrane protein that acts in conjunction with KpsE to move polysaccharide between the KpsMT cytoplasmic membrane transporter and the outer membrane (Dong et al., 2006). The genes encoding polysaccharide biosynthesis and transport by the Kps system were identified in *F. caldus*, *A. thiooxidans*, *A. ferrianus* [absence of *kps*C encoding Kdo linker (Willis, 2013)], *A. ferridurans*, and *A. ferrooxidans*. Analysis of HGT events suggested that the Kps system was from donor acidophiles, such as *kps*E, *wza*, and *kps*S genes from *Acidihalobacter*-like (Khaleque et al., 2019, 2020); *kfi*C and *kps*T from *Acidiferrobacter*-like (Issotta et al., 2018); plus *kfi*D, *kps*M, and *kps*C from Proteobacteria. Finally, the capsule formation systems Wzy and Kps were exclusively identified in *Acidithiobacillaceae* acidophiles and presented signals of HGT events from other extreme acidophiles.

**FIGURE 5:**
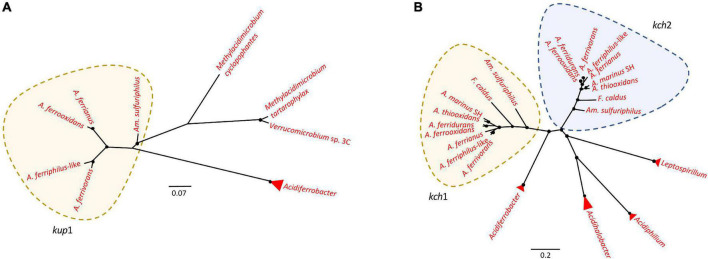
Unrooted phylogenetic tree of potassium transporters in Acidithiobacillia and their best hits in NCBI. **(A)** *kup* gene coding for Kup system had the highest similarity to homologs in extreme acidophiles *Acidiferrobacter*, *Methylacidimicrobium*, and *Verrucomicrobium*. **(B)** *kch* coding for Kch system had the highest similarity to Kch homologs in acidophiles *Leptospirillum*, *Acidiphilium*, *Acidihalobacter*, and *Acidiferrobacter*. Acidophilic sequences are highlighted in red. Filled circles at nodes represent bootstrap support of ≥60%. The scale bar represents amino acid substitution per site.

An inside positive membrane potential (Baker-Austin and Dopson, 2007; Vergara et al., 2020) suppresses the inflow of protons by creation of a chemical permeation barrier (Hu et al., 2020) and is suggested to be generated by potassium ions accumulated by Trk, Kch, Kup, and Kdo potassium channel proteins (Cholo et al., 2015; Buetti-Dinh et al., 2016; Christel et al., 2018; Wang et al., 2019). While the neutrophile *T. tepidarius* contained *trk*AH genes for TrK activity (Supplementary Figure 4), one or more of these genes were disrupted in the acidophiles *A. marinus* SH and *A. thiooxidans*. The disrupted *A. thiooxidans trk*AH genes were preceded by a NhaP antiporter and surrounded by mobile genetic elements, suggesting that they were gained by HGT events. The gene encoding NhaP antiporter, denominated *nha*P-K^+^, was classified within the first line of defense as it was in a cluster with a TrK system, suggesting a relation to potassium transport. The TrK system is a rapid-transport system at neutral or alkaline pH (Bossemeyer et al., 1989; Trchounian and Kobayashi, 2000; Epstein, 2003; Su et al., 2009) corresponding with its presence in *T. tepidarius*. Despite the disruption of the TrK system in acidophiles, the *Acidithiobacillaceae* contain three potassium transport systems that were not identified in the closest neutrophile. These were Kup (Trchounian and Kobayashi, 1999, 2000; Zakharyan and Trchounian, 2001; You et al., 2011), Kch (Voges and Jap, 1998; Munsey et al., 2002), and Kdp (Gassel et al., 1998; Yan et al., 2011; Cholo et al., 2015). The presence of three potassium transport systems in extreme acidophiles can be explained by the need to induce K^+^ uptake genes at different pH. An example is *E. coli* where the K^+^ transport TrK system is the most powerful system for K^+^ accumulation upon hyper-osmotic stress at neutral pH (Bossemeyer et al., 1989; Trchounian and Kobayashi, 2000; Epstein, 2003; Su et al., 2009; Yan et al., 2011) that is downregulated at low pH and Kup acts to compensate (Trchounian and Kobayashi, 1999; Yan et al., 2011), highlighting the role of different K^+^ uptake according to pH environment (Trchounian and Kobayashi, 1999; Yan et al., 2011). Phylogenetic analysis confirmed the similarity of Kup and Kch (Figure 5) to other extreme acidophiles such as *Acidiferrobacter* (Issotta et al., 2018), *Methylacidimicrobium* (van Teeseling et al., 2014), *Verrucomicrobium* (Schmitz et al., 2021), *Leptospirillum* (Vergara et al., 2020), and *Acidiphilium* (Li et al., 2020). This similarity was supported by HGTector prediction that suggested that Kup and Kch were transferred from Gammaproteobacteria and the acidophile *Acidihalobacter*-like. The Kup system was identified in *Am. sulfuriphilus* CJ-2 and the *Acidithiobacillus* iron oxidizers with the exception of *A. ferridurans* (Figure 5A). The Kch voltage-gated potassium channel was coded by two gene copies, *kch*1 and *kch*2, which were conserved in the *Acidithiobacillaceae* (Supplementary Figure 5). These gene copies had the highest similarity to homologs in the extreme acidophiles *Leptospirillum*, *Acidiphilium*, *Acidihalobacter*, and *Acidiferrobacter* (Figure 5B). The Kdp is a complex of four inner membrane subunits KdpF, KdpA, KdpB, and kdpC, with a KdpD sensor kinase and KdpE response regulator (Gassel et al., 1998) that are upregulated at low extracellular pH as a survival strategy for *Mycobacterium tuberculosis* (Cholo et al., 2015). The genes coding for the Kdp complex and regulatory proteins were identified exclusively in acidophiles from the class with three forms according to the gene contexts (Supplementary Figure 6). These were (i) the *kdp*CBAFED cluster conserved in *Acidithiobacillaceae* genomes except *A. marinus* SH that lacked a complete system and *Am. sulfuriphilus* with *kdp*DE genes disrupted by transposases; (ii) a second copy of the *kdp*CBAFED cluster in *Am. sulfuriphilus* and *A. ferriphilus*-like, a *kdp*ABC cluster in *F. caldus*, and *kdp*FABC in *A. ferrooxidans*; and (iii) a *kdp*ABCE gene cluster in the *F. caldus* plasmid. Mobile genetic elements were identified in the genome context of three forms of Kdp systems and BLAST analysis of Kdp encoding genes showed similarity to acidophile *Acidiferrobacter*, suggesting that the Kdp complex could be the result of an HGT event from *Acidiferrobacter*-like to *Acidithiobacillaceae* acidophiles as a mechanism to uptake potassium ions and thus improve acid resistance.

**FIGURE 6:**
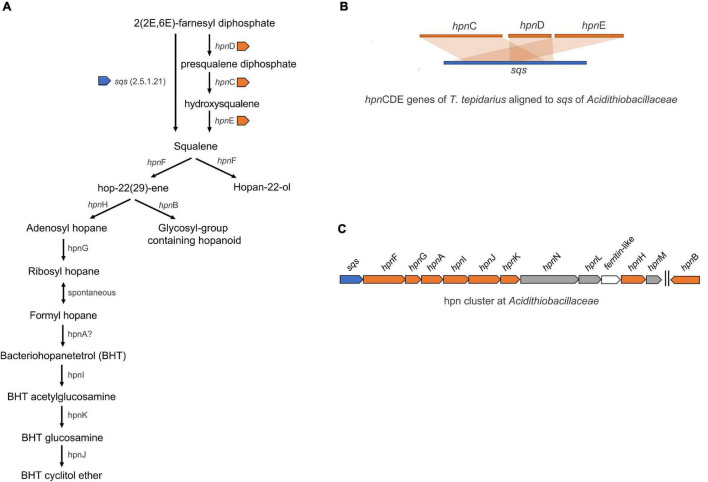
**(A)** Hopanoid biosynthesis in *Acidithiobacillaceae*. **(B)** Biosynthesis of squalene, that is required for hopanoid, is synthetized by *sqs* in acidophiles in contrast to *hpn*CDE in the neutrophile *T. tepidarius*. **(C)** The *hpn* cluster was identified in *Acidithiobacillaceae* including sqs-*hpn*FGAIJKNL-*ferritin*-*hpn*HM genes.

Comparative genomic analysis also revealed major differences between the hopanoid biosynthesis pathways of Acidithiobacillia and neutrophiles. Hopanoid biosynthesis regulates membrane fluidity, maintaining membrane integrity and permeability of cells and promoting resistance to antibiotics, detergents, extreme pH, high osmolarity (Welander et al., 2009; Wu et al., 2015), and surviving general environmental stressors such as in the cyanobacterium genus *Nostoc* (Ricci et al., 2017; Mayer et al., 2021). The neutrophile *T. tepidarius* synthesizes squalene *via* the *hpn*CDE gene products compared to the one step through *sqs* gene product in the acidophiles (Figure 6). A further hopanoid cluster was conserved in the *Acidithiobacillaceae*, including the *hpn*ABFGHIJKLMN genes conferring the ability to produce several hopanoids such as bacteriohopanetetrol (BHT), cyclitol, and hopan-22-ol. This was consistent with *A. thiooxidans* as a source of bacteriohopanepolyols (BHPs) including BHT, aminotriol, and BHT cyclitol ether (Jones et al., 2012) and the Acidithiobacillales for BHT cyclitol ether, aminotriol, BHT, and adenosylhopane at geothermal vents (Gibson et al., 2014). RNA transcripts confirmed the expression and functionality of *sqs* gene in *A. ferrivorans* (Christel et al., 2016). Phylogenomic analysis of the *hpn* cluster showed similarity with *hpn* from the acidophile *Acidiferrobacter* sp. SPIII (Supplementary Figure 7), highlighting the role of this pathway for extreme acidophiles.

### Second Line of Defense in Acidithiobacillia

**FIGURE 7:**
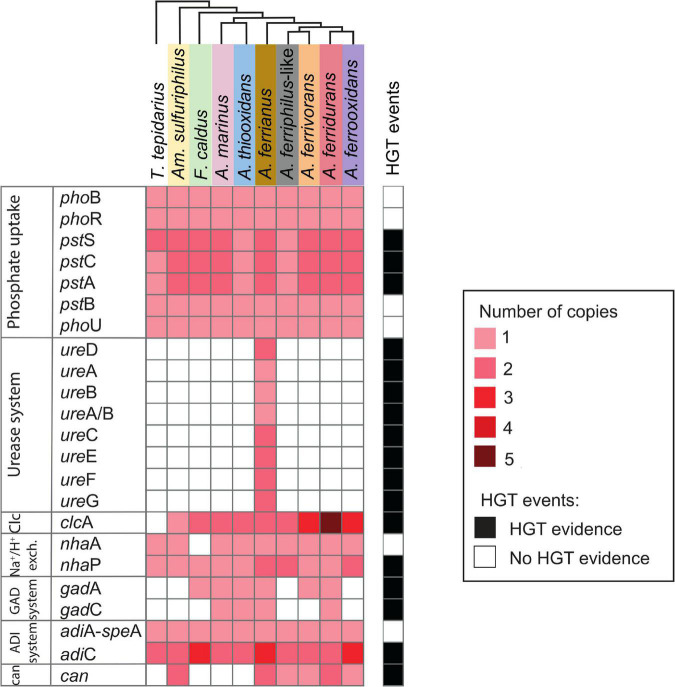
Heatmap of presence (red) and absence (white) of genes involved in second line of defense acid resistance in the Acidithiobacillia class. The red color intensity represents gene copy number per genome. The presence of genes (black; right-hand side) denotes HGT events by genome context with mobile genetic elements, phylogenetic trees, or if it was signaled as part of HGT event by HGTector (Zhu et al., 2014). Species abbreviations are as described in the Figure 3 legend. Second line of acid resistance genes include phosphate uptake, urease system, ClcA antiporter (Clc), proton antiporter (Na^+^/H^+^ exch.), amino acid decarboxylation (GAD and ADI systems), and carbonic anhydrase (can) (shaded colors correspond to those used in Figure 1).

The second line of defense includes genes coding for proton export from the cell (Figure 7). Phosphorus is of utmost importance for living organisms (Li et al., 2011) and may also ameliorate low pH stress *via* cytoplasmic buffering of protons (Baker-Austin and Dopson, 2007; Chen L. et al., 2015). The phosphate transport system genes *pst*SCAB are abundant in acidophilic *Acidithiobacillus*, *Leptospirillum*, and *Acidiphilium* taxa in acid mine drainage (Chen L. et al., 2015). A complete repertoire of phosphate uptake genes was identified in the Acidithiobacillia class including the two-component regulatory proteins PhoB/PhoR, Pst-transport system PstSCAB (Valdés et al., 2008), and auxiliary PhoU (Vuppada et al., 2018). The *Acidithiobacillaceae* genomes maintained two copies of the *pst*SCA gene cluster except *A. thiooxidans* and *A. ferriphilus*-like BY0502. HGT event signals were identified for *pst*SCA genes in *A. ferriphilus*-like BY0502 from a Bacteria donor.

**FIGURE 8:**
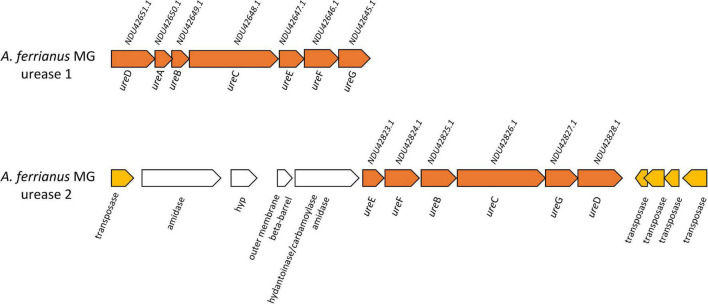
Genetic organization of urease 1 and urease 2 system in *A. ferrianus* MG. Genome context of urease 1 and extended urease 2 in *A. ferrianus* MG formed by *ure*ABCDEFG (orange) with their corresponding accession numbers. Mobile genetic elements are highlighted by yellow arrows.

The urease complex can act as buffering capacity of the intracellular pH using ammonia derived from urea hydrolysis such in *Ferrovum* group 2 strains (Ullrich et al., 2016a). The role of urease in pH homeostasis has been shown for *Helicobacter pylori* during gastric colonization (Scott et al., 1998; Stingl et al., 2002; Schoep et al., 2010) and suggested for “*Ferrovum”* strain JA12 (Ullrich et al., 2016b) and *Thiomonas* sp. CB2 (Farasin et al., 2015). The three sub-unit cytoplasmic apoenzyme urease (UreABC) synthesizes CO_2_ and NH_3_ from urea, interacting with UreDFG and UreE (Carter et al., 2009). Urease complexes were found in *A. ferrianus* MG (Figure 8) encoded by *ure*DABCEFG (urease 1) and *ure*EF(A/B)CGD (urease 2) where *ure*A and *ure*B genes were fused in a single gene denominated *ure*(A/B) as identified in *H. pylori* (Voland et al., 2003). A manual curation of genomic context of urease 2 displayed closeness with transposase elements that suggested an origin by HGT event, which was confirmed by HGTector displaying transfer of *ure*F(A/B)CG genes from Proteobacteria donors. HGT signal events were also identified for all urease 1 complex genes that were associated with Bacteria and Cyanobacteria donors. Other urease clusters, with the same gene distribution as urease 1, were identified in other *Acidithiobacillaceae* species including *A. ferrooxidans* IO-2C (new proposed annotation *A. ferridurans*), *F. caldus* BC13, and *A. ferriphilus* DSM 100412 (Supplementary Figure 8).

The Cl^–^/H^+^ antiporter ClcA prevents inner-membrane hyperpolarization at extreme acidic pH in *E. coli* (Richard and Foster, 2004) and *Bacillus coagulans* (McLaggan et al., 1990). This antiporter was gained by the extreme acidophile *Leptospirillum* as demonstrated by remaining mobile elements (Vergara et al., 2020). The acidophilic *Acidithiobacillaceae* contained two contiguous *clc*A genes with the exception of *A. ferrianus* where the genes were present in separate genomic regions (different contigs) and *Am. sulfuriphilus* that only had a single copy. In addition, the iron oxidizing species *A. ferrivorans*, *A. ferrooxidans*, and *A. ferridurans* contained more than two copies in their genomes. Phylogenetic trees indicated an association of *Acidithiobacillaceae* ClcA with other extreme acidophiles (Supplementary Figure 9) such as *Acidihalobacter prosperus*, *Ac. ferrooxydans* (Khaleque et al., 2019), *Ac. yilgarnensis* (Khaleque et al., 2020), *Thermodesulfobium acidiphilum* (Frolov et al., 2017), and *Sulfolobus acidocaldarius* (Chen et al., 2005). In addition, HGTector predicted an HGT signal from Bacteria, Proteobacteria, and acidophilic *Ferrovum*-like donors. The neutrophilic *T. tepidarius* lacked the *clc*A antiporter, which agreed with Vergara et al. (2020) who suggested that *clc*A could be gained by HGT events as a mechanism to resist extreme acid environments. A proton antiporter NhaA was identified in *T. tepidarius* and the *Acidithiobacillaceae* with the exception of *F. caldus*, which was suggested to have lost this gene according to MAUVE synteny analysis. A second proton antiporter, NhaP, was identified in the Acidithiobacillia class with additional copies in the acidophiles *A. ferrianus*, *A. ferriphilus*-like, and *A. ferrooxidans*. Finally, both *nha*P genes from *A. ferriphilus*-like were from HGT events with possible Bacteria and *Ferrovum*-like donors.

Amino acid decarboxylation systems such as glutamic acid-dependent acid resistance (GDAR) catalyze proton consumption. These systems consist of glutamic acid decarboxylases GadA/GadB enzymes and the glutamate/γ-aminobutyric acid (GABA) antiporter GadC while the arginine-dependent acid resistance (ADAR) system involves arginine decarboxylase AdiA and the arginine/agmatine antiporter AdiC (Foster, 2004; Kanjee and Houry, 2013). The GAD system encoded by *gad*A and *gad*C was identified in *A. marinus* SH, *A. thiooxidans*, *A. ferrianus*, and *A. ferridurans*. Phylogenetic analysis of GadA showed a clade of *Acidithiobacillaceae* genes sharing a common ancestor with *Acidihalobacter* acidophiles (Supplementary Figure 10), which was supported by the prediction of an HGT event from Gammaproteobacteria to *Acidithiobacillaceae* for *gad*C in *A. marinus* and *A. ferridurans*, and the presence of a mobile genetic elements (MGE) upstream of *gad*C in *A. ferrianus*. These results suggested that the GAD system was gained by acidophiles by obtention of decarboxylase GadA and amino acid permease GadC. Analysis revealed that the *F. caldus* and *A. ferrivorans* genomes only encoded *gad*A and they lacked the *gad*C antiporter (Mangold et al., 2011). However, it is proposed that the decarboxylated GABA product from glutamate decarboxylation might be retained in the cell where it can be incorporated into the TCA cycle (Karatzas et al., 2010; Feehily and Karatzas, 2013; Sriaporn et al., 2021). Even though a complete glutamate decarboxylase exporting system was not identified, previous studies have shown that amino acid decarboxylation is highly expressed in *F. caldus* under acid stress conditions (Mangold et al., 2013; Sriaporn et al., 2021). The *adi*A gene contained the domain *spe*A that also participates in putrescine synthesis and explains why this gene is associated with both pathways (*adi*A-*spe*A; Figure 7). The ADAR system was identified in both Acidithiobacillia neutrophiles and acidophiles with a single gene encoding arginine-decarboxylate AdiA and amino acid permease AdiC. However, *F. caldus*, *A. ferrianus*, and *A. ferrooxidans* had three copies of *adi*C. Finally, *adi*C genes were suggested to be acquired by HGT transfer from Proteobacteria and *Acidiferrobacter*-like donors that suggested an evolutionary gain event for acidophiles.

Carbonic anhydrase (*can*) aids pH homeostasis by catalyzing the interconversion of CO_2_ to HCO_3_^–^ (Bury-Moné et al., 2008; Valdés et al., 2008; Frost and Mckenna, 2014; Ansari and Yamaoka, 2017; Hu et al., 2020) and was identified in acidophilic microorganisms such as *Leptospirillum*, *Ferrovum* spp., *S. thermosulfidooxidans*, and *Sulfobacillus* sp. (Ullrich et al., 2016a,b; Zhang et al., 2017; Hu et al., 2020). Depending on the direction of reaction, the β-carbonic anhydrase may prevent cytoplasmic acidification by breakdown of HCO_3_^–^ (consuming H^+^) or conversion of CO_2_ to HCO_3_^–^ for carbon fixation (Lehtovirta-Morley et al., 2016). A cytoplasmic carbonic anhydrase of the β-class clade B (Valdés et al., 2008; Esparza et al., 2019) was identified in *Am. sulfuriphilus* and iron oxidizer genomes with two copies in *Am. sulfuriphilus, A. ferrianus*, and *A. ferridurans.* The additional copy of *can* in the *A. ferridurans* genome was proposed to result from a transfer event from a Bacteria donor according to HGTector. This concept was reinforced by interpretation of phylogenetic trees (clade *can*1–*can*2, Supplementary Figure 11) displaying similarity of Can from *A. ferrianus* and *Am. sulfuriphilus* with acidophiles *Acidihalobacter* and *Sulfobacillus*. Finally, a truncated *can* was found in the neutrophile *T. tepidarius*. However, its activity needs to be experimentally investigated.

### Model of *Acidithiobacillaceae* Acid Resistance

**FIGURE 9:**
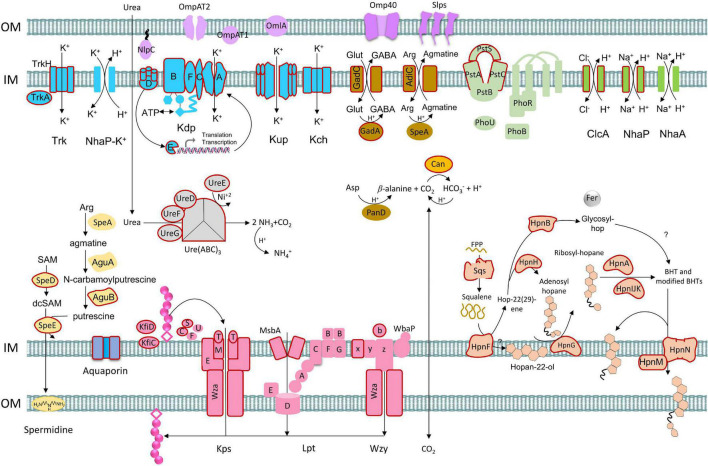
Model of acid resistance genes and mechanisms in acidophiles from the *Acidithiobacillaceae* including first and second lines of defense. Gene gain events proposed by HGT are represented with outline colors in red according to phylogenetic/phylogenomic and prediction (Zhu et al., 2014) analysis.

The model proposes the transition of a neutrophilic Acidithiobacillia ancestor to the extremely acidophilic *Acidithiobacillaceae* family by reinforcing the outer membrane and generating a positive membrane potential to restrict the influx of protons into the cytoplasm (Figure 9). Three mechanisms were exclusively identified in extreme *Acidithiobacillaceae* acidophiles, namely, (i) capsular biosynthesis pathways, (ii) influx of potassium ions, and (iii) hopanoid biosynthesis. NhaP, Kup, Kch, and Kdp were identified in acidophiles generating a transmembrane electrical potential and redundancy of genes for potassium influx. The presence of *sqs* gene in *Acidithiobacillaceae* coding for squalene biosynthesis in a single reaction (as compared with the three reactions in *T. tepidarius*) may also represent an important evolutionary energetic advantage for hopanoid biosynthesis, which, along with the *hpn*FGAIJKNLHM gene cluster, allows to synthetize and modify hopanoids. The second line in *Acidithiobacillaceae* presented a pool of accessory genes for buffering of intracellular pH, including decarboxylation of glutamate, urea hydrolysis, and hydration of CO_2_, and antiporters export excess protons by coupling the uptake of Na^+^ (Chen et al., 2021). This functional redundancy may represent a key strategy for acidophiles to live across different pH ranges and “hedge bet” in rapidly changing acidic environments, where it is hypothesized that the cost of maintaining genetic redundancy is offset by the ability to expeditiously adjust to environmental fluxes.

## Conclusion

The core genome phylogenomic and acid resistance protein phylogenetic trees confirmed the transition across the Acidithiobacillia class from an ancestral neutrophile to an extreme acidophile (Williams et al., 2010; Williams and Kelly, 2013; González et al., 2016). Acidithiobacillia acid resistance genes were classified into first and second lines of defense, where most were identified in the extremely acidophilic *Acidithiobacillaceae* family. The analysis suggested that whereas a major fraction of genes involved in acid resistance were inherited vertically, genome reprograming including duplication, gain by HGT from extreme acidophiles, and mutation of genes played a role in the evolution of the acidophilic lifestyle. Especially prominent in our analysis was the prediction of a large number of HGT events from other extreme acidophiles, suggesting that this mode of gene acquisition played a major role in the evolution of an inferred neutrophilic ancestor into a clade of extreme acidophiles.

## Data Availability Statement

The original contributions presented in the study are included in the article/Supplementary Material, further inquiries can be directed to the corresponding author.

## Author Contributions

DH and JV conceived and designed the research. CG-R performed the research. CG-R, EV, MD, JV, and DH analyzed the data. All authors participated in the writing and approval of the final manuscript.

## Conflict of Interest

The authors declare that the research was conducted in the absence of any commercial or financial relationships that could be construed as a potential conflict of interest.

## Publisher’s Note

All claims expressed in this article are solely those of the authors and do not necessarily represent those of their affiliated organizations, or those of the publisher, the editors and the reviewers. Any product that may be evaluated in this article, or claim that may be made by its manufacturer, is not guaranteed or endorsed by the publisher.

## Footnotes

1
http://tree.bio.ed.ac.uk/software/figtree/

## Supplementary Material

Supplementary Data Sheet 1 List of conserved core genes in Acidithiobacillia class.

Supplementary Data Sheet 2 List of genes involved into first and second line of defense acid resistance in the Acidithiobacillia class.

Supplementary Image 1 Supplementary figures.
