# Supplementary material for: Integrative Genomics Sheds Light on Evolutionary Forces Shaping the Acidithiobacillia Class Acidophilic Lifestyle
Source: Front Microbiol. 2022 Feb 15;12:822229. doi: 10.3389/fmicb.2021.822229 (PMC8886135; doi:10.3389/fmicb.2021.822229)
Supplement: Supplementary Image 1 — Supplementary figures. [file Image_1.pdf]

Supplementary Material

Integrative Genomics Shed Light on Evolutionary Forces Shaping the Acidithiobacillia Class Acidophilic Lifestyle

Carolina González-Rosales [1,2], Eva Vergara[1], Mark Dopson[3], Jorge Valdés[4], and David S. Holmes[1,5*]

[1]Center for Bioinformatics and Genome Biology, Centro Ciencia & Vida, Fundación Ciencia & Vida, Santiago, Chile.

[2]Center for Genomics and Bioinformatics, Faculty of Sciences, Universidad Mayor Santiago, Chile.

[3]Center for Ecology and Evolution in Microbial Model Systems (EEMiS), Linnaeus University, Kalmar, Sweden.

[4] Center for Bioinformatics and Integrative Biology, Facultad de Ciencias de la Vida, Universidad Andrés Bello, Santiago, Chile

[5] Facultad de Medicina y Ciencia, Universidad San Sebastián, Santiago, Chile.

*** Correspondence:**
David Holmes
dsholmes2000@yahoo.com

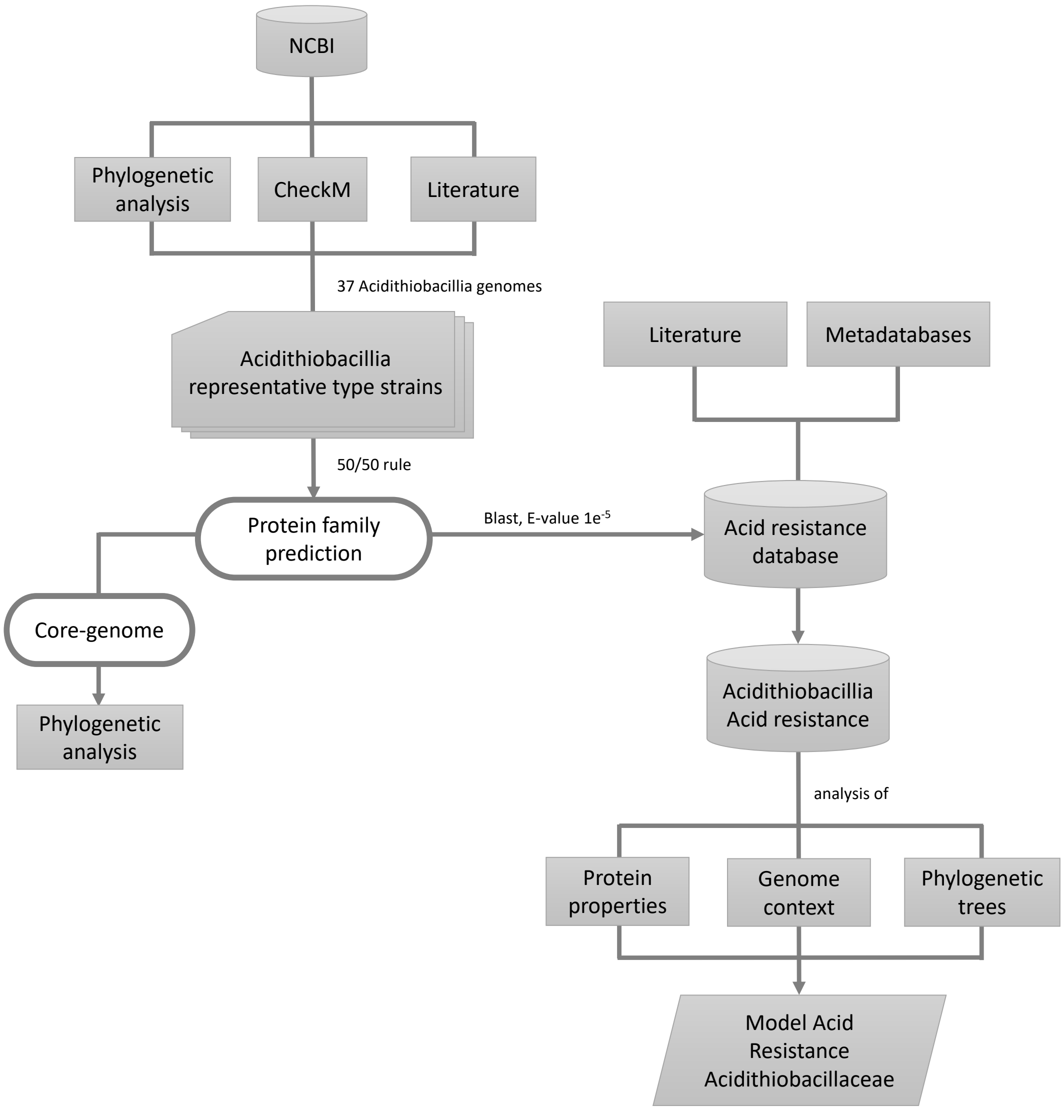

Supplementary Figure 1. Pipeline used for identifying genes/mechanisms involved in Acidithiobacillia class acid resistance.

A.

```
Tte  MKD------VLLTQQD------------LPGVLRSLFAELLGTFALTFVDAGGAVIAAV--SHDAVTLAARSAASG
Ams  M----------------------------QPMTRKMTAEFIGTFGLIFFGGGAAAMG--------KPLIDVALANG
ASH  MAENTAVKFGVKTGWR------------AQSVWSEMLAEFVGCFVLLAFGAGCVAVAVVGLTESHRTMVIFQGAGG
Ath  MAEDTSVKFGVKAGWR------------AQSVWSEMLAEFVGCFVLLAFGAGCVAVAVVGLTESHRTMVIFQGAGG
Afu  M----------------------------HQMTRRMTAEFIGTFGLIFFGGGAAAMG--------RPLIDIALANG
Afp  M----------------------------HQITRKMMAEFIGTFGLIFFGGGAAAMG--------KPLIDIALANG
Afv1 M----------------------------HQITRKMMAEFIGTFGLIFFGGGAAAMG--------KPLIDIALANG
Afv2 MTNEPRQHVVSLPERRMLHAPVPPDFLEPSHEWRRLFSEAWGTFLLVVVAAGSVVVGAW--SHGAISLSMMVVAPG
Afv3 MTNEPRQHVVSLPERRMLHAPVPPDFLEPSHEWRRLFSEAWGTFLLVVVAAGSVVVGAW--SHGAISLSMMVVAPG
Afd  M----------------------------CSISRKMAAEFIGTFGLIFFGGGAAAMG--------YPLIDVALANG
Afe  M----------------------------CSISRKMAAEFIGTFGLIFFGGGAAAMG--------YPLIDVALANG
```

B.

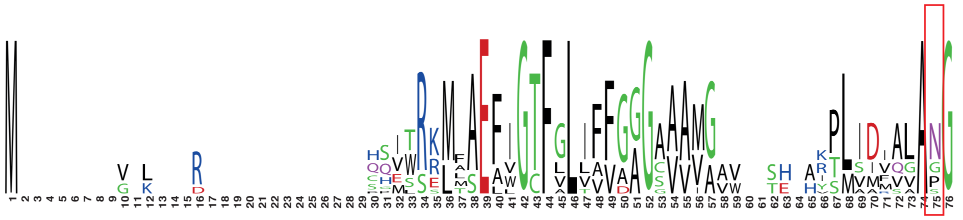

Supplementary Figure 2. Alignment of AqpF in the Acidithiobacillia. A. Mafft alignment of the first 76 AqpF amino acids with the red box showing the position 39 and in red if the amino acid is an asparagine residue (Asn39) related to acid resistance. Abbreviations: Tte-*Thermithiobacillus tepidarius* DSM 3134, Ams-*Ambacidithiobacillus sulfuriphilus* CJ-2, ASH-*Acidithiobacillus marinus* SH, Ath-*A. thiooxidans* ATCC 19377, Afu-*A. ferrianus* MG, Afp-*A. ferriphilus* BY0502, Afv1-Afv3-*A. ferrivorans* SS3 (three copies of AqpF), Afd-*A. ferridurans* JCM 18981, and Afe-*A. ferrooxidans* ATCC 23270. B. Multiple sequence alignment logos of first 76 AqpF residues.

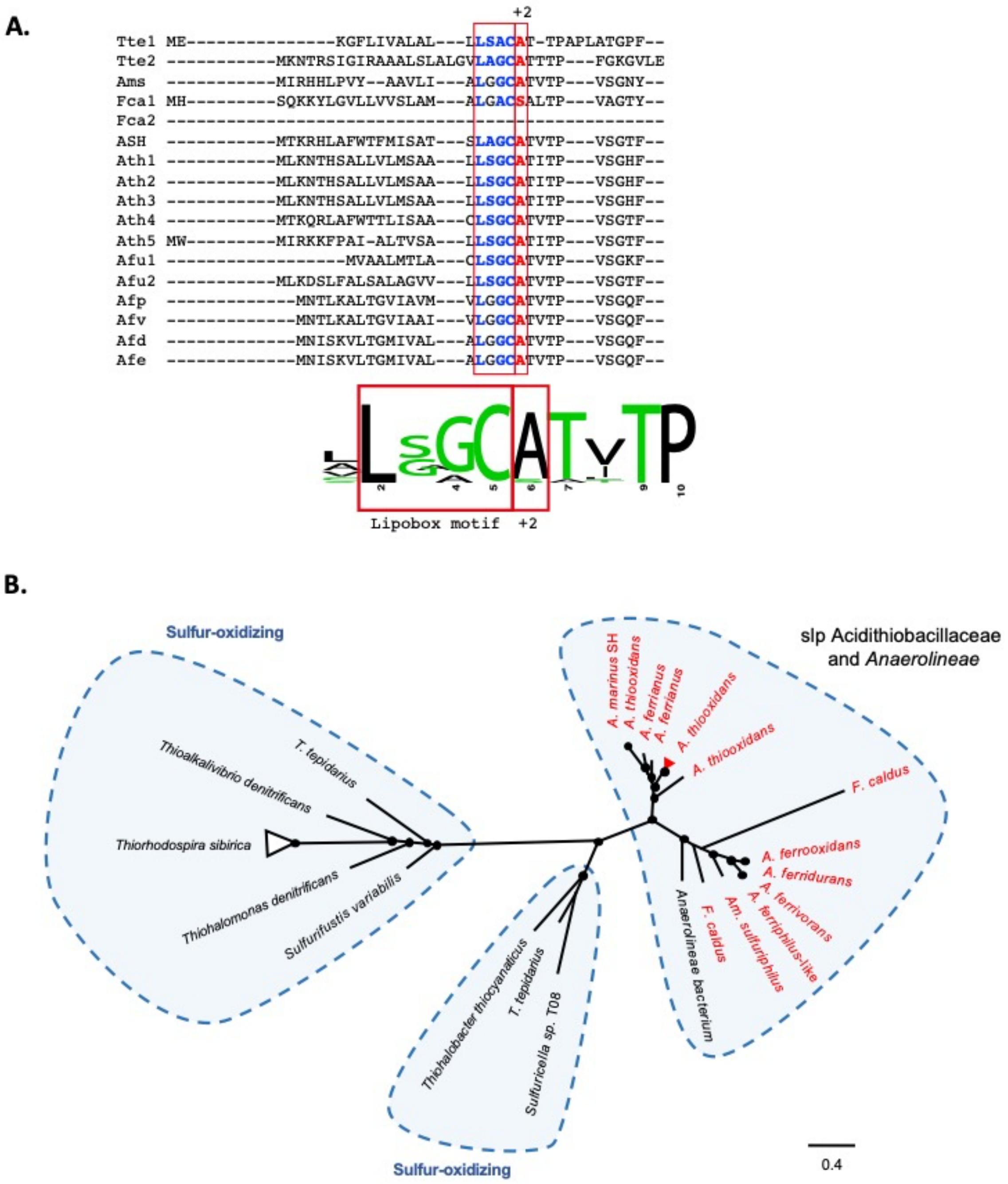

Supplementary Figure 3. Slp starvation lipoprotein. A. Mafft alignment of first 50 Slp amino acid residues in the Acidithiobacillia, highlighting the lipoprotein motif and the +2 position in the red box followed by multiple sequence alignment logos of the initial residues. Abbreviations: Tte1-2-*Thermithiobacillus tepidarius* DSM 3134, Ams-*Ambacidithiobacillus sulfuriphilus* CJ-2, Fca1-2-*Fervidacidithiobacillus caldus* ATCC 51756, ASH-*Acidithiobacillus marinus* SH, Ath1-5-*A. thiooxidans* ATCC 19377, Afu1-2-*A. ferrianus* MG, Afp-*A. ferriphilus* BY0502, Afv-*A. ferrivorans* SS3, Afd-*A. ferridurans* JCM 18981, and Afe-*A. ferrooxidans* ATCC 23270. B. Unrooted Slp amino acid sequence phylogenetic tree with their best hits in the NCBI database. Acidophilic Slp had higher similarity to homologues in *Anaerolineae* bacterium than Slp sequences from taxonomically closest

Acidithiobacillia class (*T. tepidarius*). Acidophilic sequences are highlighted with red color. Filled circles at the nodes indicate bootstrap support ≥ 60%. The scale bar represents 0.4 amino acid substitution per site.

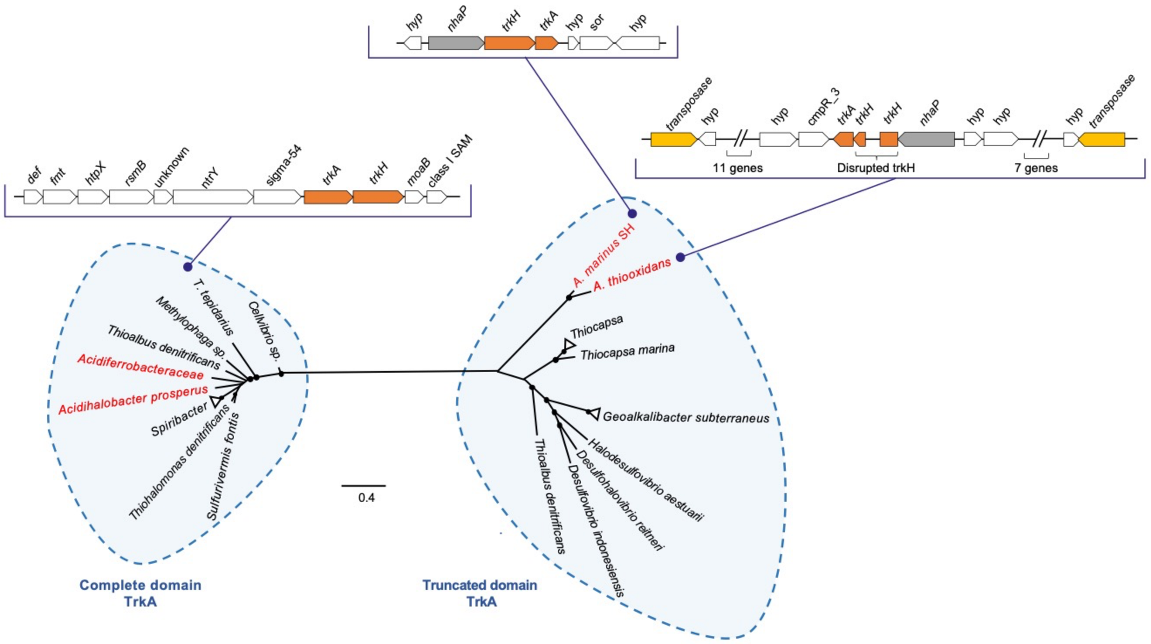

Supplementary Figure 4. Unrooted phylogenetic tree of Acidithiobacillia TrkA and their best hits in NCBI. The tree presents two main clades according to the conservation of the TrkA, a clade with species that present a complete TrkA domain and the second species including truncated domains. The Acidithiobacillaceae members shows similarities to homologues with species that have an altered TrkA domain. Acidophilic sequences are highlighted with red color. Filled circles at nodes represent bootstrap support of ≥ 60%. The scale bar represents 0.4 amino acid substitution per site. Genome context of *trk*A genes are displayed for *T. tepidarius*, *A. thiooxidans*, and *A. marinus* SH genomes. Orange arrows indicate the *trk*A and *trk*H genes.

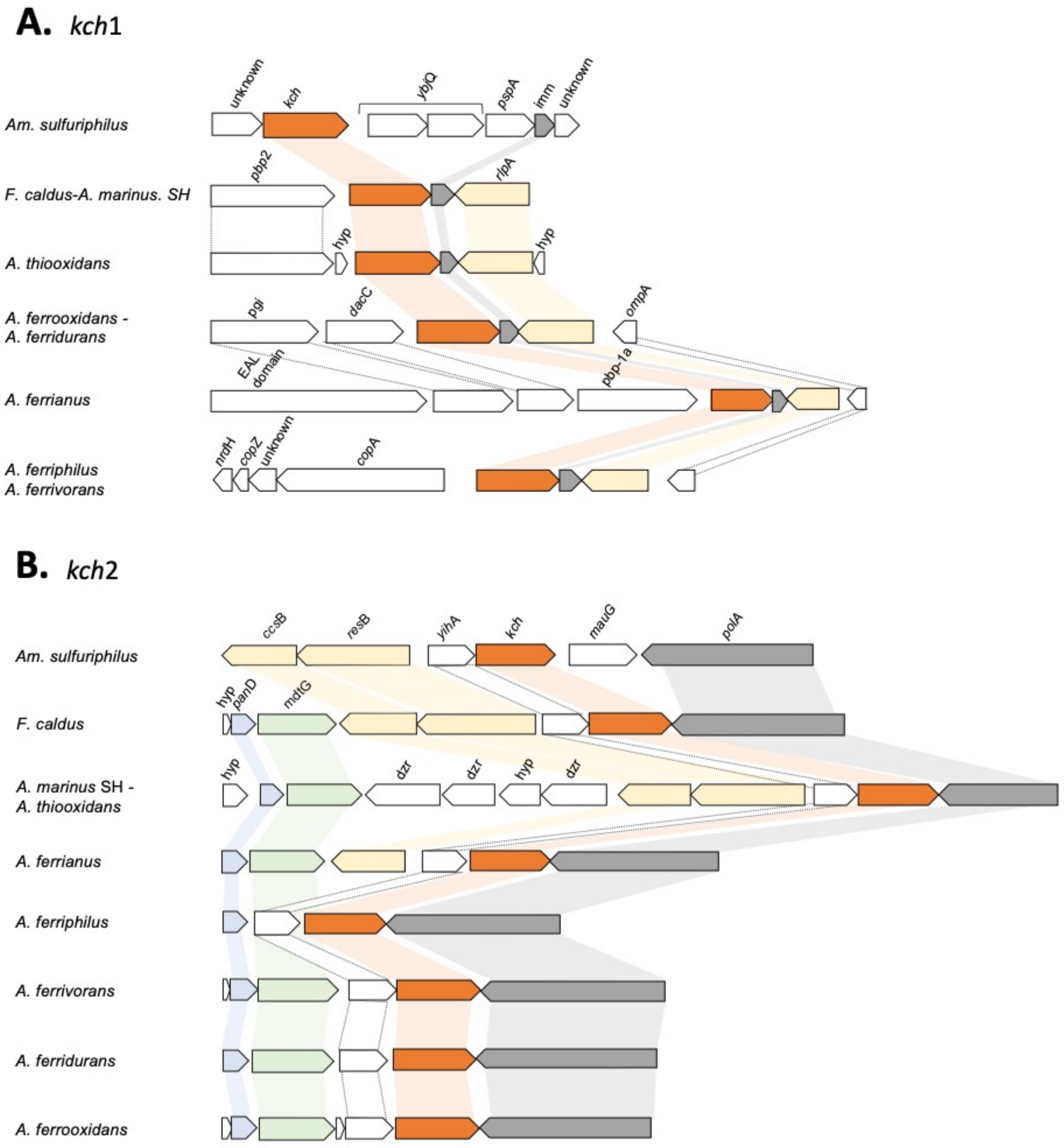

Supplementary Figure 5. Voltage-gated potassium channel Kch. A. Genome context of *kch*1. B. Genome context of *kch*2. *kch* genes are highlighted in orange.

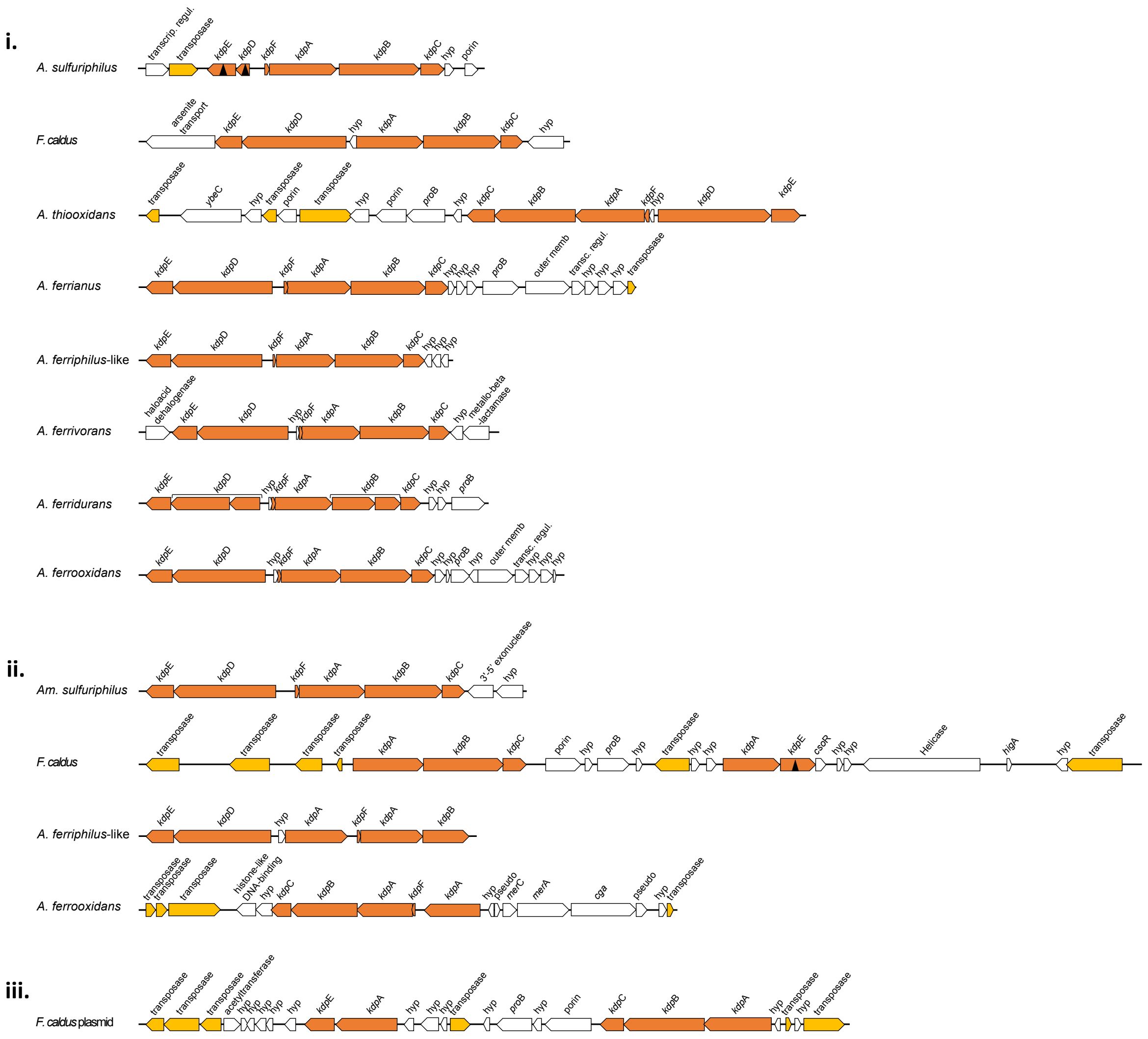

Supplementary Figure 6. Genome context of the Acidithiobacillaceae Kdp system. The Kdp system is classified in three forms according to gene contexts: i. *kdp*EDFABC in most Acidithiobacillacea. ii. Kdp systemin *Am. sulfuriphilus*, *F. caldus*, *A. ferriphilus*-like, and *A. ferrooxidans*. iii. *kdp*ABC and *kdp*E in the *F. caldus* plasmid. The genes encoding Kdp complex are represented by orange arrows and disrupted genes with black triangles.

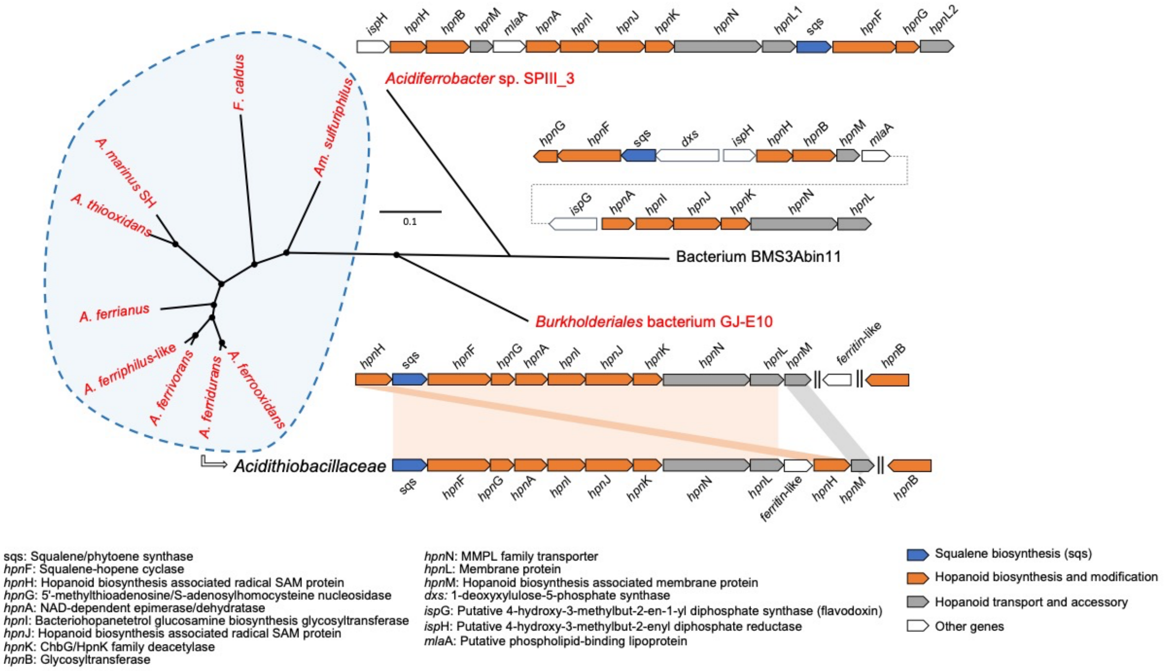

Supplementary Figure 7. Phylogenetic tree and genome context of Hpn cluster. The phylogenomic tree was constructed with concatenated amino acid sequences of *sqs*-HpnFGAIJKNLHM coding genes identified in Acithiobacillaceae and their best hits according to BLASTP comparison with NR database. The acidophilic members of Acidithiobacillia class had the highest similarity to Hpn homologues in extreme acidophiles *Acidiferrobacter* sp. SPIII_3 and *Burkholderiales* bacterium GJ-E10. Acidophiles are highlighted in red. The sequences were aligned with MAFFT with linsi option and then masked to remove unreliable regions with GBLOCKS. The concatenated alignment was used to construct the phylogenetic tree with IQTREE and LG+F+I+G as best-suited evolutionary model. Filled circles at the nodes indicate bootstrap support of ≥ 60%. The scale bar indicates number of amino acid substitutions per site.

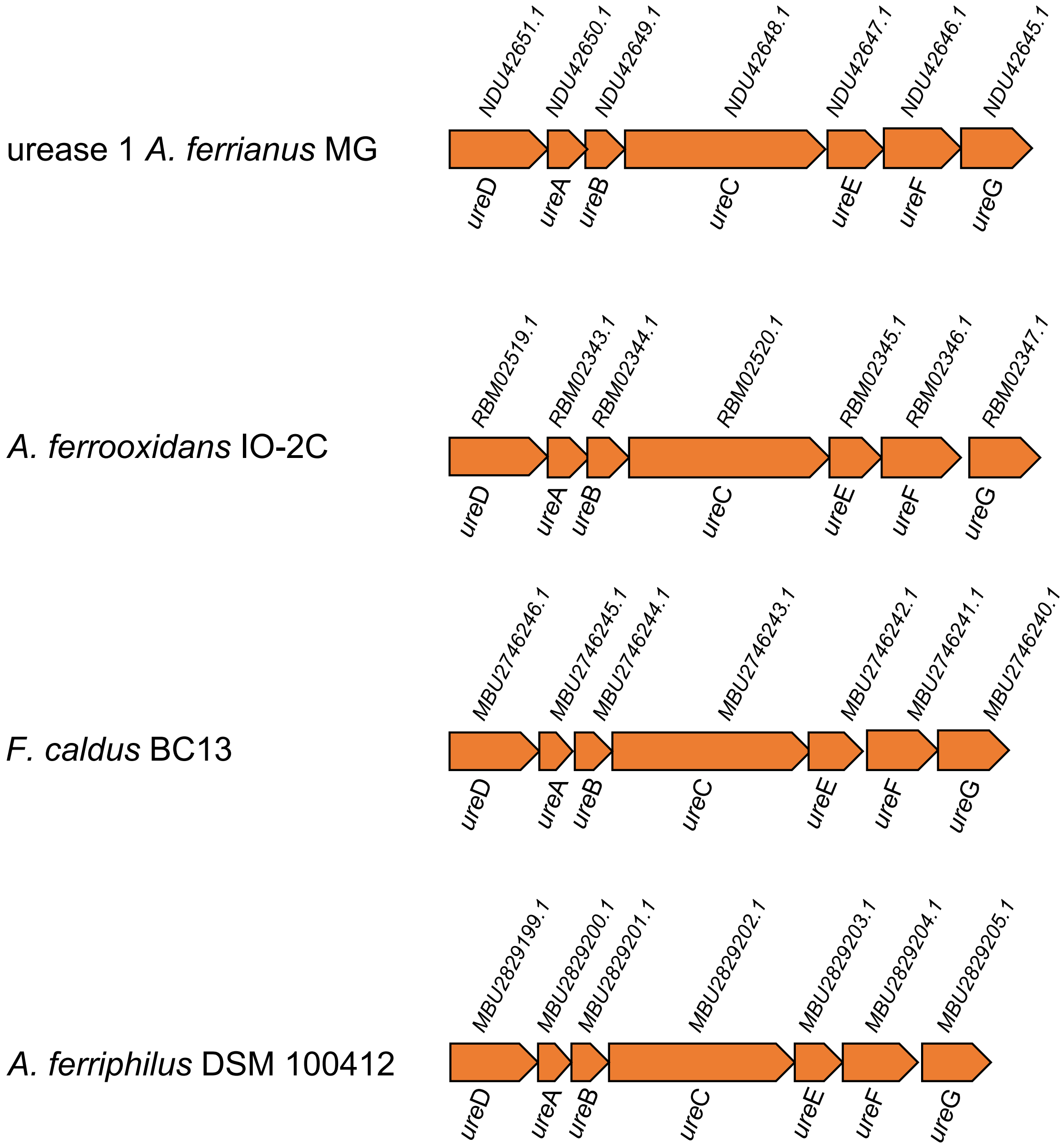

Supplementary Figure 8. Genetic organization of urease 1 from *A. ferrianus* MG and urease systems found in other non-type strains from the Acidithiobacillaceae family: *A. ferrooxidans* IO-2C, *F. caldus* BC13, and *A. ferriphilus* DSM 100412.

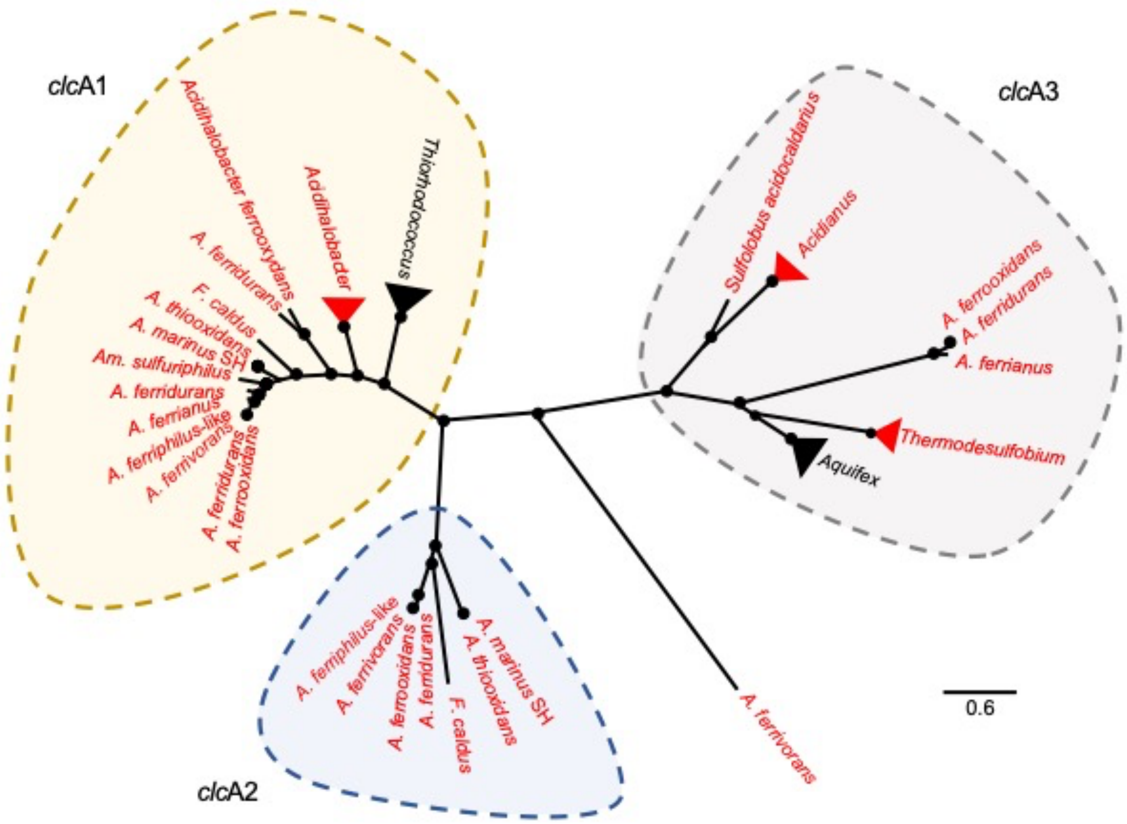

Supplementary Figure 9. Unrooted phylogenetic tree of Acidithiobacillia ClcA and their best hits in NCBI. Mostly of contiguous ClcA from Acithiobacillaceae sequences group in two *clc*A1 and *clc*A2 clades, with the exception of *A. ferrianus* sequences which had high similarity to homologues in *clc*A3 clade. The Acithiobacillaceae sequences from clade *clc*A1 show highest similarity to homologues in extreme acidophiles *Acidihalobacter*. The iron-oxidizers species, *A. ferrooxidans* and *A. ferridurans*, with a third copy of ClcA belong to clade clcA3 with highest similarity to ClcA in acidophiles *Acidianus*, *Sulfolobus acidocaldarius* and *Thermodesulfobium*. Acidophilic sequences are highlighted with red color. Filled circles at nodes represent bootstrap support of ≥ 60%. The scale bar represents 0.6 amino acid substitution per site.

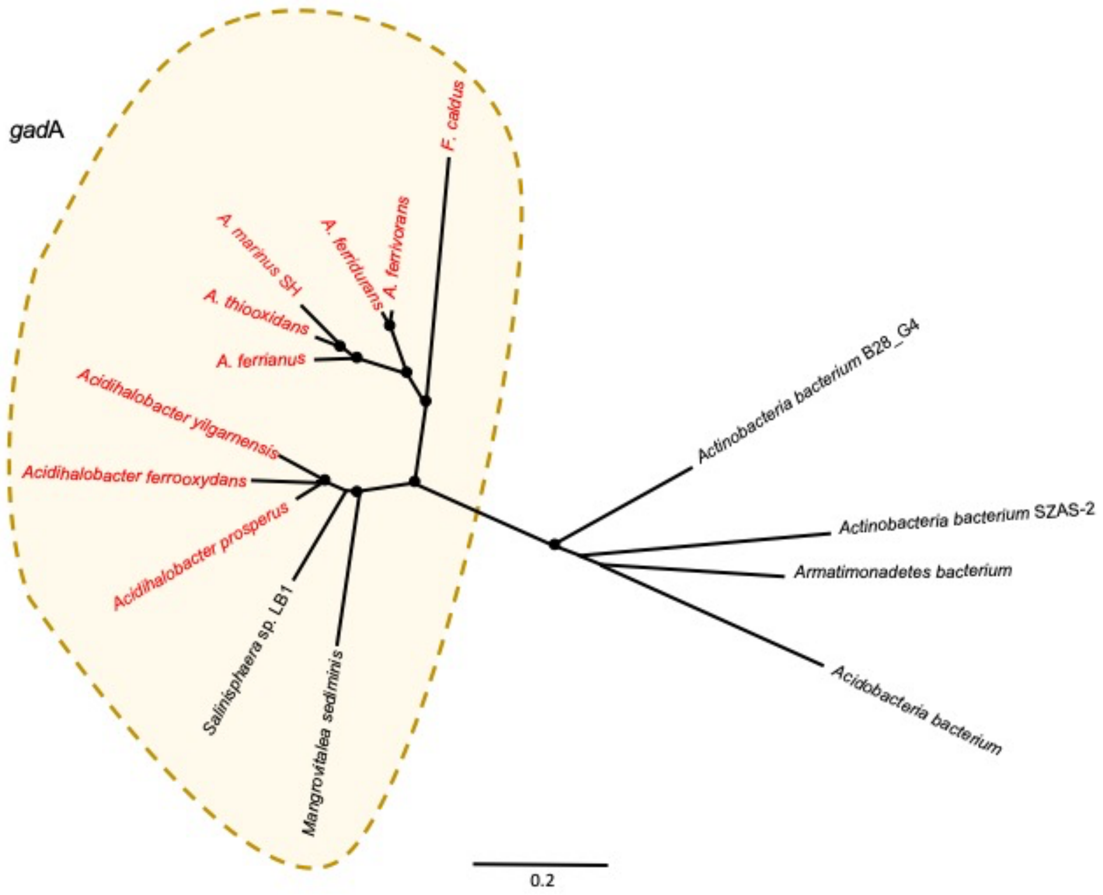

Supplementary Figure 10. Unrooted phylogenetic trees of Acidithiobacillia GadA and their best hits in NCBI. Acidithiobacillia GadA sequences had the highest similarity to homologues in extreme acidophiles *Acidihalobacter*. Acidophilic sequences are highlighted with red color. Filled circles at nodes represent bootstrap support of ≥ 60%. The scale bar represents 0.2 amino acid substitution per site.

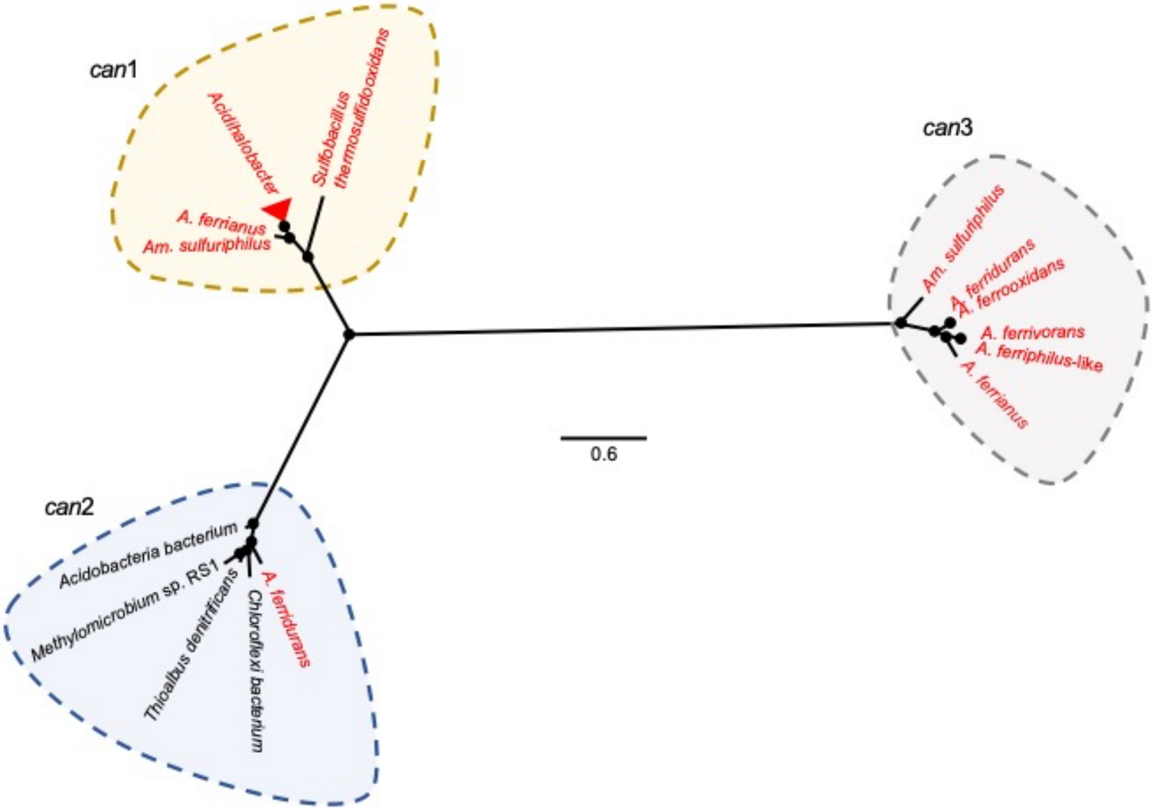

Supplementary Figure 11. Unrooted phylogenetic tree of Acidithiobacillia Can and their best hits in NCBI. Acidithiobacillia Can tree displays three clades *can*1-*can*3, meanwhile clade *can*3 collect conserved Can sequences from Acidithiobacillaceae, the clades *can*1 and *can*2 reveal high similarity between the additional copies of Acidithiobacillaceae Can with homologues in extreme acidophiles *Acidihalobacter* and *Sulfobacillus thermosulfidooxidans*. Acidophilic sequences are highlighted with red color. Filled circles at nodes represent bootstrap support of ≥ 60%. The scale bar represents 0.6 amino acid substitution per site.
